# Electromechanical Characteristic Analysis of Passive Matrix Addressing for Grating Light Modulator

**DOI:** 10.3390/s90302162

**Published:** 2009-03-24

**Authors:** Zhu Jin, Zhiyu Wen, Zhihai Zhang, Shanglian Huang

**Affiliations:** 1 The Key Laboratory of Fundamental Science of Micro/Nano Device & System Technology, Chongqing university, Chongqing, 400044, P.R. China; 2 Optoelectronic Engineering Department, Chongqing University, Chongqing, 400044, P.R. China. E-Mails: wzy@cqu.edu.cn (Z.W.); zzhincq@163.com (Z.Z.)

**Keywords:** MEMS, GLM, response frequency, driving voltage, crosstalk

## Abstract

A Grating Light Modulator (GLM) based on Micro-Electro-Mechanical Systems (MEMS) is applied in projection display. The operating principle of the GLM is introduced in this paper. The electromechanical characteristic of the passive matrix addressing GLM is studied. It was found that if the spring constant is larger, both the response frequency and the driving voltage are larger. Theoretical analysis shows that the operating voltage and the pull-in voltage of the GLM are 8.16 and 8.74 V, respectively. When an all-selected pixel in a m×n array is actuated by a voltage V_0_, the voltages of the half-selected pixel in row and column are V_0_(m–1)/(m+n–1) and V_0_(n–1)/(m+n–1), respectively, and the voltage of the non-selected pixel is V_0_/(m+n–1). Finally, the experimental results indicate that the operating voltage and the pull-in voltage are 7.8 and 8.5V respectively, and the response frequency of the GLM is about 7 kHz. The crosstalk in a 16×16 GLM array is validated by the experiment. These studies provide a theoretical basis for improving the GLM driver.

## 1. Introduction

Recently, with the development of MEMS technology, light modulators based on optical MEMS for projection displays have become a popular research focus [1–3]. There are several typical devices used in projection display such as the Digital Micro-mirror Device (DMD, [4]) invented by Texas Instruments and the Grating Light Valve (GLV, [5]) proposed by Stanford University. DMD is a precise light switch, and the mirror is rotated through the electrostatic attraction produced by the voltage difference between the mirror and the underlying memory cell. Incident light is reflected and modulated by the tilt mirror.

A DMD has a three layer structure, so its fabrication process is very complicated. A typical GLV pixel consists of an even number of parallel, dual-supported ribbons formed of silicon nitride and coated with a reflective aluminum top-layer. Initially the phase difference of GLV is zero, and the diffraction intensity of GLV is mainly focused on zero order. When the GLV is controlled by a voltage, the phase difference becomes π, and GLV will diffract the light to the first diffraction order, so a GLV can switch the light from zero diffraction order to first diffraction order by controlling the phase difference electrically. The structure of a GLV is very simple, but it is a linear array, which needs a scanning device for displaying a 2D image. This scanning device increases the complexity and the assembly difficulties of the projection system. We propose herein a novel Grating Light Modulator (GLM) [6,7] with a two layer structure. Its process is simpler than DMD, and the GLM can easily form two-dimensional modulator arrays, and with these 2D modulator arrays, the scanning device is eliminated, and the projection system can be simpler.

Currently the driving mode of the GLM array is a passive matrix addressing. This mode is very simple, and promotes the optimization of the device structure and parameters. The driver of the GLM array is a critical technology that affects the performance of the projection system. In order to improve the working performance of the GLM, the electromechanical characteristics of passive matrix addressing need to be analyzed. This paper introduces the operating principle and the fabrication process of the GLM, and analyzes its driving voltage and response frequency. Then, the crosstalk of passive matrix addressing for the GLM is analyzed. Finally, experiments are designed to measure the driving voltage and the response frequency, and validate the crosstalk in the GLM array.

## 2. The principle of GLM

**Figure 1.**
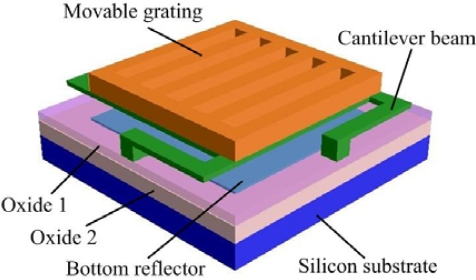
The structure of a single GLM.

Figure 1 shows the structure of a single GLM. It is consisted of silicon substrate, bottom reflector, silicon dioxide, cantilever beam and movable grating.

Both the movable grating and the bottom reflector are made of aluminum, and they are separated by an insulating layer and an air gap. The movable grating and the bottom reflector form a phase grating, and the phase difference is determined by the space between the movable grating and the bottom reflector.

**Figure 2.**
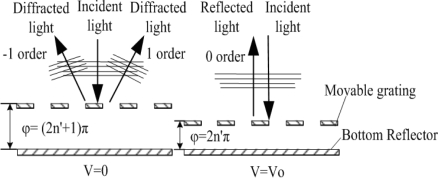
The operating principle of the GLM.

**Figure 3.**
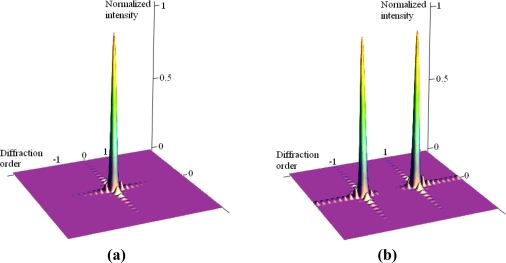
The diffraction intensity distribution of the GLM. **(a)** The phase difference is 2n'π. **(b)** The phase difference is (2n'+1)π.

In Figure 2, when no voltage is applied between the movable grating and the bottom reflector, the phase difference of the GLM equals to (2n'+1)π (n' is a positive integer), and the diffraction light is mainly focused on the ±1^st^ order. When a voltage is actuated between them, the electrostatic force pulls the movable grating downwards. The phase difference equals to 2n'π, and the diffraction light is mainly focused on the zero order. The switch between the dark state and the bright state can be realized by collecting the zero order or the ±1^st^ order diffraction light. Figure 3 shows the diffraction intensity distributions of the GLM when the phase difference is 2n'π or (2n'+1)π.

**Figure 4.**
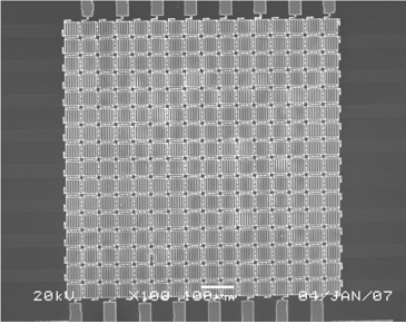
A SEM photograph of passive matrix addressing for the 16×16 GLM array.

Passive matrix addressing for the GLM is composed of a horizontal electrode group and a vertical electrode group. Every GLM’s bottom reflector in a row connects to a horizontal electrode which produces a row pulse signal, and every GLM’s movable grating in a column connects to a vertical electrode which is taken as a data line. When all horizontal electrodes are scanned by the progressive mode, a frame image can be displayed by a GLM array. Figure 4 shows a SEM photograph of the fabricated 16×16 GLM array addressed by passive matrix.

## 3. Device fabrication

**Figure 5.**
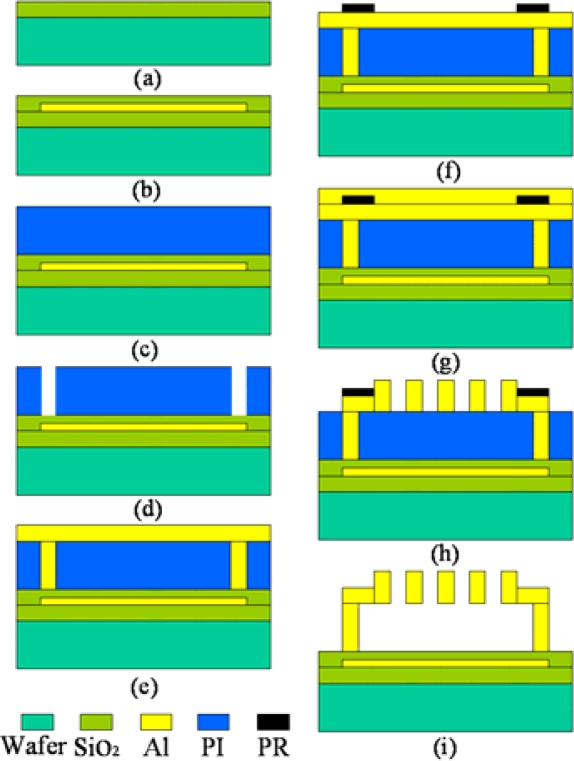
The fabrication process of GLM.

The GLM fabrication process [8] is shown in Figure 5. The fabrication begins with a <100> p-type silicon wafer made using surface micro-machining technology. For electrical isolation among bottom electrode, 600 nm thick thermal silicon dioxide is deposited. Then 100 nm aluminum is sputtered and patterned to make the bottom reflector. Next, 280 nm silicon dioxide was deposited as a dielectric layer by PECVD.

**Figure 6.**
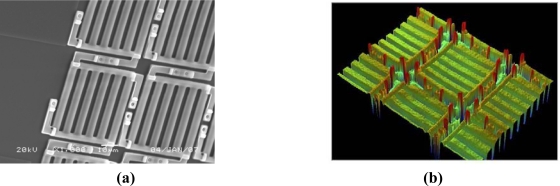
The photograph of the fabricated GLM array. **(a)** The SEM photograph of parts of a GLM array. **(b)** The VEECO photograph of parts of the fabricated GLM array.

After the formation of SiO_2_ layer, 580 nm polyimide (PI) is spin-coated as a sacrificial layer, and four support post cavities are etched by plasma on the sacrificial layer. Then 200 nm aluminum is sputtered, and four support cavities are filled with aluminum. Then, the photoresist (PR) is spin-coated as a protection layer and exposed, and another 530 nm aluminum is sputtered. Two aluminum layers are lithographed to form the upper movable grating, and the aluminum under PR is formed to cantilever beam. Finally, both PI and PR are etched by plasma at the same time. Figure 6a shows the SEM photograph of one corner of the fabricated 16×16 array. The width of the movable grating ribbon is 4 μm, the grating constant of the movable grating is 8μm, and the size of a single GLM is 52 × 52 µm. The residual stress σ of the cantilever beam measured by the MEMS foundry is equal to about 100 Mpa[8]. Figure 6b is a photograph of parts of the GLM array by VEECO. Figure 6 indicates that the surface of the movable grating is quite smooth.

## 4. Theoretical analysis of the GLM’s electromechanical characteristics

**Figure 7.**
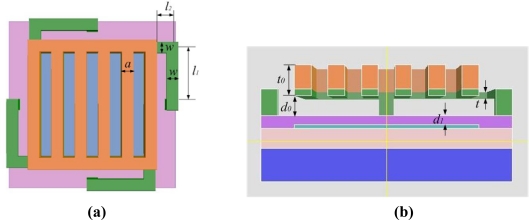
The structure and the parameters of the GLM. **(**a) The top image; (b) The cross-section image.

The motion of the movable grating is determined by the electrostatic force and the mechanical restoring force. The movable grating and the bottom reflector can be considered as a variable plane-parallel capacitor. The thickness of the cantilever beam using a buried-beam process is quite thin. The thickness of the movable grating is much thicker than the cantilever beam, as shown in Figure 7, where l_1_ and l_2_ are the two part lengths of the cantilever beam, t is the thickness of the cantilever beam, w is the width of the cantilever beam, d_0_ is the initial space between the movable grating and the dielectric layer, and d_1_ is the thickness of the dielectric layer. When the GLM is actuated by a voltage, the movable grating will pull down rigidly. The movable grating can be considered as a rigid body, so the spring constant of the movable grating can be ignored. A cantilever beam is taken as a spring, and the four cantilever beams are equivalent to the four shunt springs. Therefore, the GLM can be described as the capacitor-spring model.

The response frequency which affects the grayscale of the passive matrix GLM is a key parameter. The faster the response frequency is, the larger the grayscale is. The driving voltage is also a key parameter for the passive matrix GLM, and the lower driving voltage can decrease the complexity and power in system control circuits. The fabricated GLM should have high response frequency and low driving voltage characteristics. Furthermore, the passive matrix addressing for the GLM results to the crosstalk which will reduce the contrast and the light utilization of the GLM.

### 4.1. Response frequency and driving voltage

The resonant frequency w_0_ equals to $\sqrt{k/m}$ [10], where m is the effective mass of the movable grating and k is the spring constant of the GLM. If the spring constant is larger, the response frequency is faster. The driving voltage of the GLM is obtained as [8]:

(1) $$V=(d_{0}+\frac{d_{1}}{\varepsilon_{1}}-y)\sqrt{\frac{2\mathrm{ky}}{\varepsilon \varepsilon_{0}\gamma A}}$$

where A is the area of the movable grating, y is the displacement of the movable grating, γ is a correctional factor, ε_1_ is the relative dielectric constant of the dielectric layer, and ε which is the relative dielectric constant of the air equals to 1.

The initial phase difference of the designed GLM is (2n'+1)π. When a voltage is applied on the GLM, the electrostatic force pulls the movable grating down. If y is λ/4(λ is the wavelength of incident light), the phase difference becomes 2n'π, and this driving voltage is called the operating voltage of the GLM. If y is $\frac{1}{3}(d_{0}+d_{1}/\varepsilon_{1})$, the electrostatic force is much larger than the mechanical restoring force, the movable grating will pull in, and this driving voltage is called the pull-in voltage of the GLM. According to the analysis above, it is indicated that the spring constant of the GLM is larger, and both the response frequency and the driving voltage are larger. In equation (1), the spring constant k of the GLM is only an unknown variable, so it is necessary to analyze the spring constant of the GLM.

The spring constant k’ of a cantilever beam which is determined by the structure parameter and the material characteristic of the cantilever beam is composed of two spring constants. k’ is the sum of k_1_ and k_2_. Where k_1_ results from the stiffness of the cantilever beam, and k_2_ is caused by the residual stress. First, the spring constant k_1_ can be written as [9, 10]:

(2) $$k_{1}=\frac{\mathrm{Ew}{\left(\frac{t}{l_{2}}\right)}^{3}}{1+\frac{l_{1}}{l_{2}}\left[{\left(\frac{l_{1}}{l_{2}}\right)}^{2}+12\frac{1+v}{1+{\left(\frac{w}{t}\right)}^{2}}\right]}$$

where σ is the residual stress of the cantilever beam, v is Poisson ratio, and E is Young modulus. Furthermore, the spring constant k_2_ can be obtained as [11]:

(3) $$k_{2}=\frac{\sigma (1-v)tw}{2(l_{1}+l_{2})}$$

Finally, because of four shunt cantilever beams, the spring constant of the GLM is the sum of the four cantilever beams’ spring constants, and it can be expressed as follows:

(4) $$k=4(k_{1}+k_{2})=\frac{4\mathrm{Ew}{\left(\frac{t}{l_{2}}\right)}^{3}}{1+\frac{l_{1}}{l_{2}}\left[{\left(\frac{l_{1}}{l_{2}}\right)}^{2}+12\frac{1+v}{1+{\left(\frac{w}{t}\right)}^{2}}\right]}+\frac{2\sigma (1-v)tw}{\left(l_{1}+l_{2}\right)}$$

**Figure 8.**
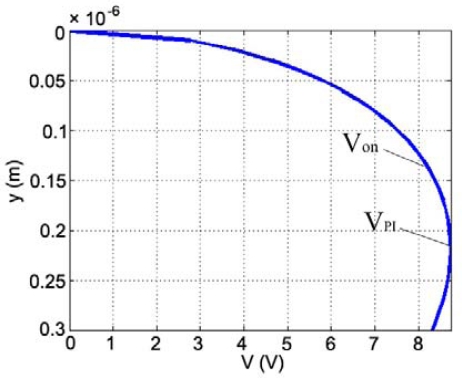
The simulation of the displacement and the driving voltage.

According to equation (4) and Table 1, k equals to 16.1758 N/m. Substitute the correlative parameters into equation (1), the relationship of the displacement y and the driving voltage V can be calculated, as shown in Figure 8. When y is 133 nm (λ/4, λ=532 nm), the operating voltage V_on_ is 8.16 V; when y is 217.3 nm ( $\frac{1}{3}(d_{0}+d_{1}/\varepsilon_{1})$), and the pull-in voltage V_PI_ is 8.74 V.

### 4.2. Analysis of crosstalk in the GLM array

The GLM can be equivalent to a capacitor C_g_ which can be expressed as follows [8]:

(5) $$C_{g}=\frac{\varepsilon \varepsilon_{0}A}{h}\left[1+\frac{h}{\pi a}+\frac{h}{\pi a}\text{ln}\left(\frac{2\pi a}{h}\right)+\frac{h}{\pi a}\text{ln}\left(1+\frac{2t_{0}}{h}+2\sqrt{\frac{t_{0}}{h}+\frac{t_{0}{}^{2}}{h^{2}}}\right)\right]$$

where a is the width of the movable grating ribbon, t_0_ is the thickness of the movable grating as shown in Figure 7, and h is the equivalent distance which equals to (d_0_+d_1_/ε_1_−y).

**Figure 9.**
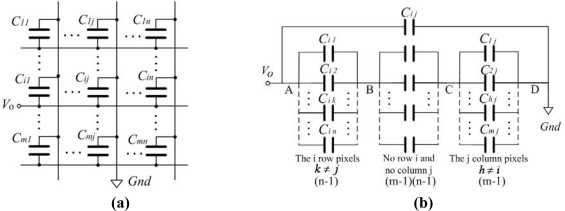
The matrix capacitance model of a m×n GLM array. (a) The initial model. (b) The simplified model.

A passive matrix addressing for the GLM causes the matrix capacitance’s coupling effect which is also called crosstalk. In a m×n GLM array, a voltage V_0_ is applied on the cross pixel of the i row and the j column, and this cross pixel is called all-selected pixel, as shown in Figure 9a. The other pixels in the i row and the j column are called half-selected pixel, and the rest pixels in the array are called non-selected pixel.

It’s supposed that the initial voltages of all pixels are zero. By using Kirchhoff’s current law(KCL)and Kirchhoff’s voltage law(KVL)[15], Figure 9b can be simplified from Figure 9a. Where V_AB_ is the voltage of the half-selected pixel in the all-selected pixel row, V_CD_ is the voltage of the half-selected pixel in the all-selected pixel column, and V_BC_ is the voltage of the non-selected pixel. According to Figure 9b, the relationship of the different voltages can be obtained as:

(6) $$\{\begin{array}{l} V_{\mathrm{BC}}\;(t)=\frac{C_{\mathrm{AB}}}{C_{\mathrm{BC}}}V_{\mathrm{AB}}\;(t)\\ V_{\mathrm{CD}}\;(t)=\frac{C_{\mathrm{AB}}}{C_{\mathrm{CD}}}V_{\mathrm{AB}}\;(t)\\ V_{\mathrm{AB}}+V_{\mathrm{BC}}+V_{\mathrm{CD}}=V_{0} \end{array}$$

Every GLM is the same in the GLM array, so the capacitance value of every GLM is the same:

(7) $$\{\begin{array}{l} C_{\mathrm{AB}}=(n-1)\cdot C_{g}\\ C_{\mathrm{BC}}=(m-1)\cdot (n-1)\cdot C_{g}\\ C_{\mathrm{CD}}=(n-1)\cdot C_{g} \end{array}$$

Taking equation (7) into (6), the voltages of the different pixels can be given by:

(8) $$\{\begin{array}{l} V_{\mathrm{AB}}=\frac{m-1}{m+n-1}V_{0}\\ V_{\mathrm{CD}}=\frac{n-1}{m+n-1}V_{0}\\ V_{\mathrm{BC}}=\frac{1}{m+n-1}V_{0} \end{array}$$

From equation (8), its shown that the voltage of the half-selected pixel is smaller than that of the total-select pixel, but its much bigger than that of the non-selected pixel. If the GLM array is larger, the crosstalk is more serious. As the 16×16 array, the voltages of the half-selected pixel and the non-selected pixel are 15/31(about 1/2) and 1/31 times respectively of the voltage of the total-select pixel.

A single GLM is actuated by a voltage, which results in the crosstalk in the GLM array. When the GLM array is scanned by the progressive mode, there may be lots of pixels which are actuated by the different voltages at the same time. It can be analyzed by the same method as before. The voltages of the half-selected pixel and the non-selected pixel may be different, but the crosstalk is more obvious. So the GLM array addressed by passive matrix can be applied in projection display with a low resolution and a low definition.

## 5. Experiments

**Figure 10.**
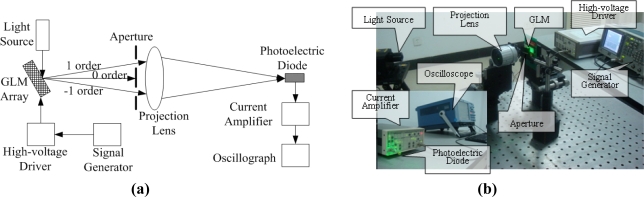
The experimental system. (a) The sketch diagram of the experimental system. (b) The actual experimental system.

Figure 10 shows the testing system of the passive matrix addressing for the GLM array. It is consisted of a light source, a 16×16 GLM array, a high-voltage driver, a signal generator, an aperture, a projection lens, a photoelectric diode, a current amplifier and an oscilloscope. The 16×16 GLM array is controlled by the high-voltage driver and the signal generator. A green laser is diffracted by the GLM array, and only the ±1^st^ order diffraction light is passed by the aperture. Then, the ±1^st^ order diffraction light passes the projection lens and displays an image of the GLM array. Only a single GLM pixel enters the photoelectric diode. The light intensity of the ±1^st^ order diffraction can be converted to a photocurrent by the photoelectric diode. This photocurrent is amplified and converted to a voltage signal by the current amplifier, and this voltage signal is displayed on the oscilloscope.

### 5.1. Results of driving voltage and response frequency

**Figure 11.**
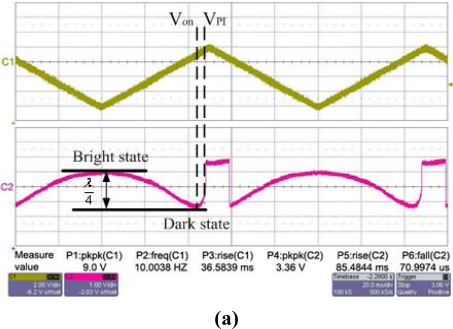

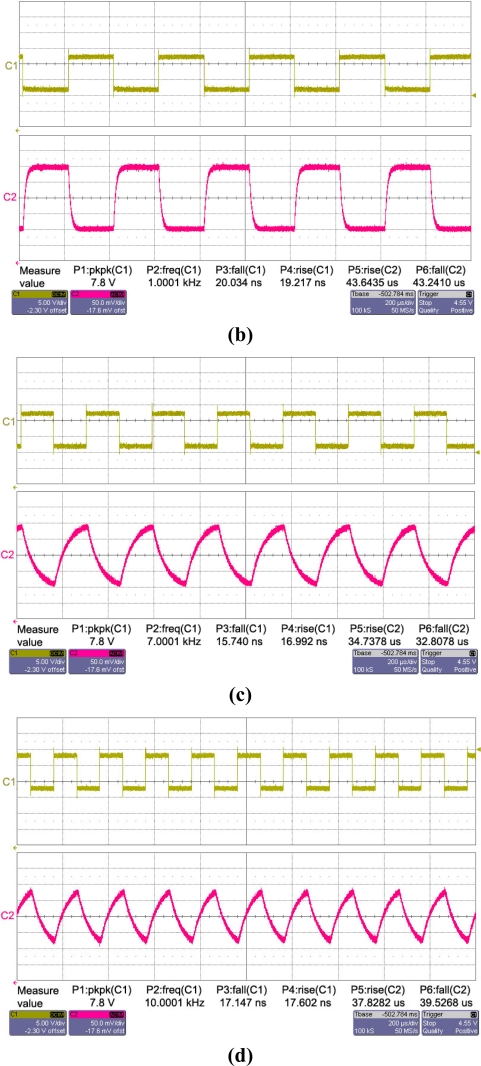
The testing results of the ±1^st^ order diffraction light. (a) The intensity response of the GLM actuated by a ramp-wave. (b) The intensity response of the GLM actuated by a square-wave of 7.8 V and 1 kHz frequency. (c) The intensity response of the GLM actuated by a square-wave of 7.8 V and 7 kHz frequency. (d) The intensity response of the GLM actuated by a square-wave of 7.8 V and 10 kHz frequency.

In Figure 11, the upper curve “C1” is the driving voltage of the GLM, and the bottom curve “C2” is the ±1^st^ order intensity response of the GLM. Figure 11a shows that the GLM is actuated by a ramp-wave voltage. The ±1^st^ order initial phase difference of the fabricated GLM is (2n'+1)π. According to the operating principle of the GLM, when no voltage is applied, the intensity of the ±1^st^ order diffraction light is the bright state, and a high voltage signal is read out on the oscilloscope. When the ramp-wave voltage increases slowly, the movable grating moves down slowly, the intensity of the ±1^st^ order diffraction light will decrease, and the voltage signal of the ±1^st^ order diffraction light on the oscilloscope reduces. When the movable grating moves down a λ/4 distance, the phase difference become 2n'π, and the light intensity is in a minimum which is the dark state. As a result of the phase’s period, the ramp-wave voltage continues to increase, the light intensity increases, but as the movable grating pulls in, the light intensity jumps to a maximum. Figure 11a shows that the operating voltage V_on_ of the GLM is 7.8 V and the pull-in voltage V_PI_ of the GLM is 8.5 V, which are very close to the theoretical values. The difference between the theoretical value and the experimental value results from the slight difference between the designed structure and the fabricated structure.

Figures 11b, 11c and 11d are the ±1^st^ order light intensity responses of the GLM actuated by the square-wave voltages of 7.8 V and different frequencies. For a half period, no voltage is actuated on the GLM, and the light intensity is in the maximum. For the other half period, a 7.8 V voltage is actuated on the GLM, and the light intensity is in the minimum. Figure 11b shows that the GLM works well at 1 kHz frequency, and the rise time and the fall time of the intensity response are about 43.64 μs and 43.24 μs, respectively. Figure 11c indicates that when the frequency is 7 kHz, the intensity response wave is distorted. And when the frequency is 10 kHz, the intensity response wave gets attenuated obviously, as seen in Figure 11d. The results indicate that the response frequency of the GLM is about 7 kHz.

### 5.2. Results of the crosstalk

**Figure 12.**
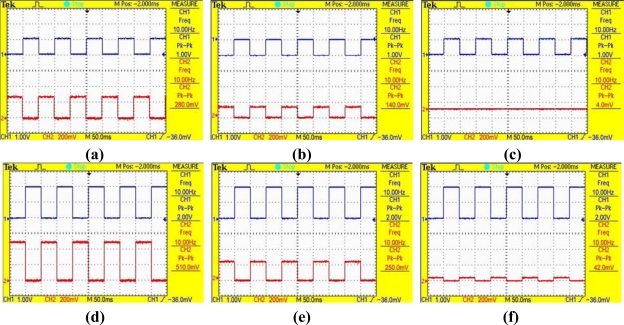
The ±1^st^ order diffraction light intensities of the different pixels when an all-selected pixel is actuated by different square-waves. (a) all-selected pixel when actuated by a 1V square-wave. (b) half-selected pixel when an all-selected pixel is actuated by a 1V square-wave. (c) non-selected pixel when an all-selected pixel is actuated by a 1V square-wave. (d) all-selected pixel when actuated by a 2 V square-wave. (e) half-selected pixel when an all-selected pixel is actuated by a 2V square-wave. (f) non-selected pixel when an all-selected pixel is actuated by a 2V square-wave.

In Figure 12, the upper curve “1” is the square-wave voltage of the all-selected pixel, and the bottom curve “2” is the ±1^st^ order diffraction light intensity of the different pixels. As the fabrication processes of all pixels in array are the same, the pixels with the same peak-to-peak value of the diffraction light intensity have the same actuated voltage in the same scope of the phase difference. By comparing Figure 12a with Figure 12e, the peak-to-peak value of the light intensity is 280 mV when the square-wave voltage of the all-selected pixel is 1V, which is very approximate to 250 mV when the square-wave voltage of the all-selected pixel is 2 V. It can be concluded that the square-wave voltage of the half-selected pixel in Figure 12e is about 1V, which is consistent to the theory that the voltage of the half-selected pixel is half of the all-selected pixel in the 16×16 GLM array. The results also show that when an all-selected pixel is actuated by a voltage, the voltage of the half-selected pixel is much larger than the voltage of the non-selected pixel, but it is smaller the voltage of the all-selected pixel, which agrees with the crosstalk theory of the matrix capacitance.

**Figure 13.**
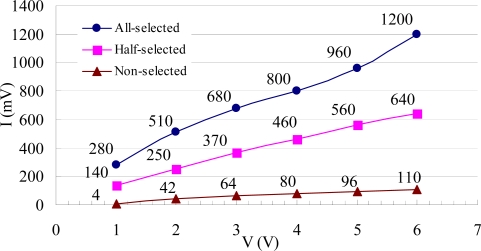
The ±1^st^ order diffraction light intensity of different pixels when an all-selected pixel is actuated by 1V to 6V square-waves voltages.

The ±1^st^ order diffraction intensities of the different pixels when an all-selected pixel is actuated by 1 V to 6 V square-wave voltages are shown in Figure 13. The results indicate that the peak-to-peak values of those three pixels all increase when the voltage of the all-selected pixel increases. Using the same analysis as before, it is shown that in the 16×16 GLM array the voltage of the half-selected pixel is half of the all-selected pixel, and the non-selected pixel voltage is quite small, which also agrees with the theoretical analysis.

## 6. Conclusions

The operating theory of passive matrix addressing for GLM is introduced in this paper. The response frequency and the driving voltage of the GLM are analyzed, and the crosstalk which influences the performance of the GLM array is also studied in detail. It is concluded that: (1) both the response frequency and the driving voltage are related to the spring constant of the GLM. The theoretical operating voltage and the pull-in voltage are 8.16 V and 8.74 V respectively; (2) in a GLM array, the half-selected pixel voltage is half of the all-selected pixel in an array, and the voltage of the half-selected pixel is much larger than that of the non-selected pixel. With the increase of the GLM array, the crosstalk becomes more serious; (3) the experimental results indicate that the operating voltage and the pull-in voltage are 7.8 V and 8.5 V, respectively, very close to their theoretical values. When the GLM is actuated by a square-wave voltage of 7.8 V and 1 kHz frequency, the rise and the fall times of the intensity response are about 43.64 μs and 43.24 μs, respectively. The response frequency of the GLM is about 7 kHz. Another experiment confirms the crosstalk in the GLM array. A GLM array addressed by passive matrix can be applied in low resolution and low definition projection displays. These studies can provide a theoretical basis for improving the driving characteristic of the GLM. In future, in order to eliminate the crosstalk of passive matrix addressing for GLM, we are planning to use an active matrix method in driving the GLM array, which can be applied in projection display with high resolution and high definition.

## Figures and Tables

**Table 1.** The correlative parameters of the designed GLM [8].

**d_1_ (μm)**	**ε_1_**	**ε_0_ (F/m)**	**d_0_ (μm)**	**γ**	**A (μm^2^)**	**t (μm)**	**w (μm)**	**l_1_ (μm)**	**l_2_ (μm)**	**E (Gpa)**	**v**	**σ (Mpa)**
0.28	3.9	8.854 × 10^−12^	0.58	1.28	1533	0.2	4.0	10.5	3.5	77	0.3	100
